# Cytogenetic identification and molecular marker development for the novel stripe rust-resistant wheat–*Thinopyrum intermedium* translocation line WTT11

**DOI:** 10.1007/s42994-021-00060-3

**Published:** 2021-10-11

**Authors:** Guotang Yang, Qi Zheng, Pan Hu, Hongwei Li, Qiaoling Luo, Bin Li, Zhensheng Li

**Affiliations:** 1 State Key Laboratory of Plant Cell and Chromosome Engineering, Institute of Genetics and Developmental Biology, The Innovative Academy of Seed Design, Chinese Academy of Sciences, Beijing, 100101 China; 2 University of Chinese Academy of Sciences, Beijing, 100049 China

**Keywords:** Wheat–*Thinopyrum intermedium* translocation line, CYR34, Cytogenetic analyses, Wheat660k SNP array, PCR-based markers, KASP markers

## Abstract

Stripe rust, caused by *Puccinia striiformis* f. sp. *tritici* (*Pst*), is one of the most destructive diseases of wheat (*Triticum aestivum* L.) worldwide. Xiaoyan 78829, a partial amphidiploid developed by crossing common wheat with *Thinopyrum intermedium*, is immune to wheat stripe rust. To transfer the resistance gene of this excellent germplasm resource to wheat, the translocation line WTT11 was produced by pollen irradiation and assessed for immunity to stripe rust races CYR32, CYR33 and CYR34. A novel stripe rust-resistance locus derived from *Th. intermedium* was confirmed by linkage and diagnostic marker analyses. Molecular cytogenetic analyses revealed that WTT11 carries a TTh·2DL translocation. The breakpoint of 1B was located at 95.5 MB, and the alien segments were found to be homoeologous to wheat-group chromosomes 6 and 7 according to a wheat660K single-nucleotide polymorphism (SNP) array analysis. Ten previously developed PCR-based markers were confirmed to rapidly trace the alien segments of WTT11, and 20 kompetitive allele-specific PCR (KASP) markers were developed to enable genotyping of *Th. intermedium* and common wheat. Evaluation of agronomic traits in two consecutive crop seasons uncovered some favorable agronomic traits in WTT11, such as lower plant height and longer main panicles, that may be applicable to wheat improvement. As a novel genetic resource, the new resistance locus may be useful for wheat disease-resistance breeding.

**Supplementary Information:**

The online version contains supplementary material available at 10.1007/s42994-021-00060-3.

## Introduction

Wheat stripe rust, caused by *Puccinia striiformis* f. sp. *tritici* (*Pst*), is one of the factors limiting wheat production in most wheat-growing regions subject to cool and moist weather conditions (Chen et al. 2014). Historically, the breeding of resistant varieties has been considered to be an economical and environmentally friendly method to control stripe rust. Although more than 80 stripe rust-resistance genes have been identified and cataloged to date, only a few have been researched in depth (Zhang et al. 2019a). For example, *Yr5*, *Yr7* and *YrSP* are located on wheat chromosome 2B and encode nucleotide-binding and leucine-rich repeat proteins (NLRs) possessing a non-canonical N-terminal zinc-finger BED domain (Marchal et al. 2018). *Yr15*, mapped onto chromosome 1B, encodes a putative kinase-pseudokinase protein (Klymiuk et al. 2018). *Yr36*, discovered in wild emmer wheat, includes kinase and putative START lipid-binding domains (Fu et al. 2009). *Yr18/Lr34* on wheat chromosome 7D encodes adenosine triphosphate-binding cassette (ABC) transporters (Krattinger et al. 2009). *Yr46/Lr67*, which has been mapped onto wheat chromosome 4D, encodes a predicted hexose transporter, and *YrAS2388*, derived from *Aegilops tauschii*, encodes a typical nucleotide oligomerization domain-like receptor (NLR) (Moore et al. 2015; Zhang et al. 2019a). *YrU1* is derived from the diploid wheat *Triticum urartu* and encodes a coiled-coil-NBS-leucine-rich repeat protein with N-terminal ankyrin-repeat and C-terminal WRKY domains (Wang et al. 2020a). Despite current research progress, the stripe rust pathogen mutates faster than the breeding of disease-resistant varieties and is thus a threat to future world food security (Li 2010). As an example, CYR34 (V26), a new pathogenic race of *Pst* virulent to *Yr24/Yr26* and *Yr36*, was first documented in 2008 (Zeng et al. 2015; McIntosh et al. 2018). The percentage of wheat stripe rust infections attributed to CYR34 increased sharply, from 0 to 34.85%, during 2009–2016, and CYR34 is now a predominant strain in Gansu Province, China (Huang et al. 2018). Therefore, the exploration of new resistant germplasm resources and the application of their resistance genes in wheat genetic improvement is thus crucial.

Secondary and tertiary gene pools of common wheat constitute a large reservoir of potentially valuable genes for enriching wheat genetic diversity (Bommineni and Jauhar 1997). A total of 18 stripe rust-resistance genes have been currently documented in these two gene pools: *Yr8*, *Yr17*, *Yr19*, *Yr28*, *Yr37*, *Yr38*, *Yr40*, *Yr42* and *Yr70* from *Aegilops* (Riley et al. 1968; Bariana and McIntosh 1993; Chen et al. 1995; Singh et al. 2000; Marais et al. 2005a, 2006, 2009; Kuraparthy et al. 2007; Bansal et al. 2016); *Yr7*, *Yr24* and *Yr53* from *Triticum durum* (Macer 1963; McIntosh and Lagudah 2000; Xu et al. 2013); *Yr15*, *Yr35* and *Yr36* from *Triticum dicoccoides* (McIntosh et al. 1996; Marais et al. 2005b; Chicaiza et al. 2006); *Yr9* and *Yr83* from *Secale cereal* (Zeller 1973; Li et al. 2020); and *Yr50* putatively derived from *Thinopyrum intermedium* (Host) Barkworth and D. R. Dewey [syn. = *Agropyron intermedium* (Host) Beauvoir = *Elytrigia intermedia* (Host) Nevski] (Liu et al. 2013). *Th. intermedium* (2*n* = 6*x* = 42) possesses many favorable features, including genes conferring resistance against wheat streak mosaic virus, rust, powdery mildew and scab (Friebe et al. 1996; Han et al. 2003; Fedak and Han, 2005; Qi et al. 2007; He et al. 2009; Luo et al. 2009). Since *Th. intermedium* is immune to stripe rust, it can be widely applied for wheat-resistance improvement (Bao et al. 2014).

As important intermediates produced by the backcrossing of common wheat with wheat–relative hybrids, partial amphiploids generally carry the complete wheat genome in addition to a set of alien chromosomes (Fedak et al. 2000). The wheat–*Th. intermedium* partial amphiploids Zhong 1–7, TAF46, TAI7047, TAI8335, TE253-I, TE257, TE346 and Xiaoyan 78829 have been successively created (Qi et al. 1979; Banks et al. 1993; Zhang et al. 1996; Fedak et al. 2000; Chang et al. 2010; Bao et al. 2014). Among them, Zhong 2, Zhong 4, Zhong 5, TAI7047, TAI8335, Xiaoyan 78829, TAF46, TE253-I, TE257 and TE346 are immune or highly resistant to stripe rust. Using partial amphiploids as intermediate parents is of both theoretical and practical significance in wheat chromosome engineering breeding. The wheat–*Th. intermedium* substitution line W44 and translocation lines such as Z4, CH13-21, CH4131 and CH4132 with good stripe rust resistance have been developed by crossing wheat–*Th. intermedium* partial amphiploids with common wheat. The resistance of all these new materials is believed to be derived from *Th. intermedium* (Friebe et al. 1992; Larkin et al. 1995; Zhan et al. 2015; Zheng et al. 2020). Among these lines, W44 has been determined to be a 7Ai-2(7D) substitution line by C-banding and is nearly immune to stripe rust. Z4, which has a stable chromosome number of 44 and has displayed effective stripe rust resistance for over 40 years, contains two pairs of non-Robertsonian translocations—TrI and TrII—and lacks wheat chromosome 3A. Evaluation of the stripe rust resistance of Z4 and its derived progenies confirmed adult plant resistance (APR) to stripe rust comes from TrII (Lang et al. 2018). CH13-21 is derived from a cross between the wheat–*Th. intermedium* partial amphiploid TAI7047 and the common wheat line Mianyang 11. Genomic in situ hybridization (GISH), multi-color fluorescence in situ hybridization (mc-FISH) and multi-color GISH (mc-GISH) have demonstrated that CH13-21 contains 40 wheat chromosomes and a pair of T6BS.6Ai#1L compensating Robertsonian translocation chromosomes. At adult plant stage, CH13-21 is highly resistant to *Pst* races CYR30, CYR32 and CYR33. Two wheat–*Th. intermedium* translocation lines CH4131 and CH4132 were screened with highly resistance to *Pst* races at adult plant stage, whose translocated chromosome configuration was T3Ai-1BS.1BL. Its alien chromosome segments belonged to group 3 chromatin using sequence characterized amplified region (SCAR) and intron targeting (IT) markers’ analyses.

In a previous, unpublished study, we found that Xiaoyan 78829 is nearly immune to *Pst* races. Our research group has since been focused on the development of more wheat–*Th. intermedium* translocation lines with good agronomic traits as well as disease resistance. In the present study, we developed a novel translocation line, WTT11, with excellent stripe rust resistance and agronomic traits. The aims of this study were to (1) characterize the translocation line WTT11 using cytogenetic methods and a wheat660K single-nucleotide polymorphism (SNP) array; (2) validate the resistance of alien segments to *Pst* race CYR34 by linkage and diagnostic marker analyses; (3) develop PCR-based markers located on the alien chromosome fragments; (4) develop kompetitive allele-specific PCR (KASP) markers enable genotyping of *Th. intermedium* and common wheat and (5) evaluate the agronomic performance of WTT11 during two consecutive growing seasons. WTT11 broadens the wheat-resistance gene pool and can be used as a novel germplasm resource for wheat-resistance breeding.

## Materials and methods

### Plant materials and *Pst* race

**Fig. 1:**
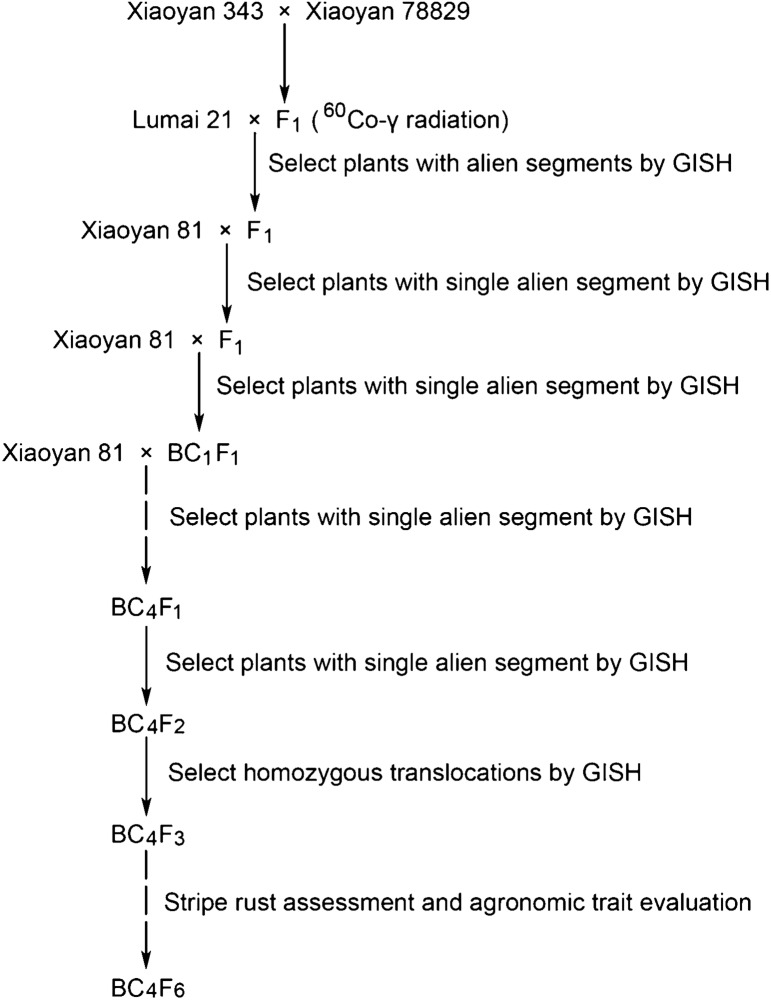
Procedure for producing wheat–*Th*. *intermedium* translocation line WTT11

WTT11 is a wheat–*Th. intermedium* BC_4_F_6_ translocation line with the pedigree Xiaoyan 81*5/3/Lumai 21//Xiaoyan 343/Xiaoyan 78829 (Fig. 1). First, Xiaoyan 343 (AABBDD, 2*n* = 6*x* = 42) was hand pollinated with pollen of Xiaoyan 78829 (AABBDDEE, 2*n* = 8*x* = 56) to produce F_1_ interspecific hybrids. The ^60^Co γ-ray-irradiated pollens of F_1_ interspecific hybrids were pollinated to elite common wheat cultivar Lumai 21. Selected translocation lines were then backcrossed five times with the recurrent parent Xiaoyan 81. Finally, homozygous translocation lines were selected from self-bred seeds. Seedling disease assessment was carried out on BC_4_F_4_ plants. Totally, 121 BC_2_F_1_ individuals from population Y11 (2) (Yannong 19*3/WTT11) were used for linkage analysis. Nineteen *Th. intermedium* accessions provided by the USDA-ARS Western Regional Plant Introduction Station, Pullman, WA, USA, were used for wheat660K SNP array analysis and KASP marker development and are detailed in Table S1. All materials are preserved in the laboratory of Zhensheng Li, Institute of Genetics and Developmental Biology (IGDB), Innovative Academy of Seed Design, Chinese Academy of Sciences (CAS), Beijing, China. The *Pst* race CYR32, CYR33 and CYR34 was provided by the stripe rust assessment platform of the IGDB, CAS.

### Chromosome preparation

Chromosome spreads were prepared according to Han et al. (2006) with minor modifications. In brief, three seeds of each line were germinated on moist filter paper in a 23 °C incubator for 2 days. Root tips with a length of 1–2 cm were collected, pretreated with N_2_O under 10 atm pressure for 2 h, fixed in 90% acetic acid for 8 min and then immediately digested with cellulase and pectinase. The meristem of root tips was mashed and diluted with 100% acetic acid. Finally, 10 μL of this mixture was dropped onto the center of a slide. Slides with good mitotic phases were used in subsequent analyses. Chromosome spreads were prepared according to the above procedure from each BC_2_F_1_ individual from Y11 (2) population.

### GISH, mc-FISH and mc-GISH analyses

GISH, mc-FISH and mc-GISH were performed following the procedures of Han et al. (2009) with some modification. For the GISH analysis, *Th. intermedium* genomic DNA (gDNA) labeled with fluorescein-12-dUTP (green) served as a probe, while Chinese Spring gDNA was used as a block. The ratio of probe to block was 1:200. The two probes used for mc-FISH, pAs1 and pSc119.2, were labeled with Texas-red-5-dCTP (red) and fluorescein-12-dUTP (green), respectively. For mc-GISH, fluorescein-12-dUTP (green)-labeled *Th. intermedium* and *T. urartu* gDNAs and Texas-red-5-dCTP (red)-labeled *Ae. tauschii* gDNA were used as probes, and *Ae. speltoides* gDNA was used as a block. The mc-GISH analysis was used to identify A-, B-, D- and *Th. intermedium* genomes. After hybridization, slides were washed in 2 × Saline Sodium Citrate and then counterstained with 4, 6-diamidino-2-phenylindole. Cells with clear hybridization signals were photographed with a DP80 CCD camera attached to an Olympus BX53 microscope and analyzed using the program CellSens Standard 1.12 (Olympus, Tokyo, Japan).

### Stripe rust-resistance evaluation

When first leaves were fully expanded, 10 individuals of WTT11 and its parents were inoculated with a mix of urediniospores and talc. The inoculated seedlings were subsequently kept in a dew and darkness chamber at 10 °C for 24 h and then transferred to a greenhouse under 70% relative humidity and 14 h of light at 16 °C and 10 h of darkness at 14 °C. When the susceptible controls were heavily infected, 2 weeks after inoculation, plant infection types (ITs) were recorded on a 0–4 scale, where 0 and 0; represent immunity and near immunity, respectively, and 1–4 represent progressively higher susceptibility, as evidenced by increasing sporulation and decreasing necrosis or chlorosis. Plants with IT scores of 0–2 were regarded as resistant, whereas those with scores of 3–4 were considered susceptible (Kang et al. 2017). The recurrent parents Xiaoyan 81 and Yannong 19 were used as susceptible control cultivars, and 121 individuals from the Y11 (2) population were also tested for seedling resistance.

### Molecular marker analysis

To confirm whether WTT11 contained any known resistance genes in *Th. intermedium*, two simple sequence repeat (SSR) markers including *Xbarc1096* for detecting *Yr50* (Liu et al. 2013), *Xbarc187-1B-1* for *YrL693* (Huang et al. 2014) and one SCAR marker *D05-A4-1* for *YrCH-1BS* (Zheng et al. 2020) were amplified in WTT11 and its parents. The sequence information and annealing temperatures of these markers are listed in Table S2. The PCR amplification volume contained of 12.5-μL 2 × Taq PCR Master Mix (CoWin Biosciences, Beijing, China), 1 μL of DNA template (100 ng/μL), 1 μL of each primer (10 μM) and 9.5 μL of ddH_2_O. The PCR cycling protocols were as follows: one cycle at 94 °C for 3 min for denaturation; 30 cycles at 94 °C for 30 s, 48–54 °C (depending on annealing temperature for each primer) for 30 s, 72 °C for 30 s; one cycle at 72 °C for 5 min for final extension. The PCR amplification products were separated in a 2% agarose gel and photographed with the Tanon 1600 Gel Image System (Tanon, Shanghai, China).

### Wheat660K SNP array analysis

To explore deletion events and homoeology between alien segments and wheat chromosomes, extracted gDNAs of WTT11 and three replicates of Xiaoyan 81 and *Th. intermedium* (PI401208) were genotyped using the 660 K wheat Affymetrix Axiom SNP array following the standard procedure of the CapitalBio Technology Company (Beijing, China). SNPs were classified according to performance metrics, such as call rate and number of minor alleles, into six categories: Poly High Resolution, No Minor Homozygote, Mono High Resolution, Call Rate Below Threshold, Off-Target Variant and Other. All SNPs with unique physical positions on the WTT11 genome were collected to analyze deletion events. The deletion rate for each chromosome of WTT11 was calculated in 3-MB sliding windows, with 1-MB steps. Except for marker-deficient regions, those regions in which the deletion ratio was statistically higher than the average value for a given chromosome were considered to have experienced deletion events. Genotypes of wheat chromosomes of WTT11 should theoretically have been similar to those of Xiaoyan 81, as WTT11 was backcrossed to Xiaoyan 81 five times and then selfed six times, whereas alien DNA sequences of WTT11, being derived from the wheatgrass chromosomes in Xiaoyan 78829, was expected to be consistent with *Th. intermedium* genomic sequences. SNPs showing homozygosity and pleomorphism between Xiaoyan 81 and *Th. intermedium*, especially those belonging to Poly High Resolution and No Minor Homozygote categories, were used to analyze the homoeology of alien segments and wheat chromosomes. The ratio of heterozygous SNPs of WTT11 originating from *Th. intermedium* and Xiaoyan 81 were counted in 50-MB sliding windows, with 1-MB steps. The alien segments of WTT11 were homoeologous to the proportion of wheat chromosomes with highest heterozygosity.

### Verification of specific markers

Using specific-locus amplified fragment (SLAF) sequencing technology, 10 previously developed PCR-based markers (*M-XNXY68-4*, *M-XNXY68-33*, *M-XNXY68-41*, *M-XNXY68-50*, *M-XNXY68-86*, *M-XNXY68-100*, *M-XNXY68-151*, *M-XNXY68-353*, *M-XNXY68-361*, and *M-XNXY68-390*) were confirmed to be specifically amplified in DT11 (denominated as WTT11) and *Th. intermedium* (Yang et al. 2019). Total gDNA of 121 individuals of BC_2_F_1_ population Y11 (2) were extracted using the sodium N-dodecanoylsalcosinate method. The 10 markers were PCR amplified in the Y11 (2) population in reaction volumes consisting of 17-μL Green Mix (Tsingke Biological Technology, Beijing, China), 1 μL of template DNA (100 ng/μL) and 1 μL of each primer (10 μM). The PCR cycling protocol was as follows: an initial step of 98 °C for 2 min, followed by 35 cycles of 98° C for 10 s, 50–60 °C (i.e., the appropriate annealing temperature for each marker) for 15 s, and 72 °C for 10 s, with a final extension of 72 °C for 5 min.

### Development and validation of KASP markers

Some SNPs on the Axiom Wheat-Relative Genotyping Array were recently converted to KASP markers (Grewal et al. 2020a). To develop KASP markers in this study, a slightly modified method was used. All flanking sequences of specific SNPs on the 660 K wheat Affymetrix Axiom SNP array from WTT11 alien segments were used in a BLASTN search against the wheat reference sequence (IWGSC RefSeq v1.0; IWGSC et al. 2018). Only sequences with a single BLAST hit to a contig were retained. KASP markers based on the 120-bp sequence surrounding the target SNP were then designed using online software (https://galaxy.triticeaetoolbox.org/).

To validate the specificity and stability of the developed KASP markers, we genotyped 19 accessions of *Th. intermedium* and 6 wheat cultivars (Xiaoyan 343, Lumai 21, Xiaoyan 81, Yannong 19, Jimai 22 and Chinese Spring). KASP marker amplifications were performed in 10-μL reaction volumes consisting of 5 μL of 2 × KASP master mix, 0.14-μL primer assay mix, 1-μL template DNA (50 ng/μL) and 3.86-μL ddH_2_O on an ABI StepOnePlus instrument. PCR cycling conditions were as follows: an initial step of 94 °C for 15 min and 94 °C for 20 s, followed by 10 touchdown cycles of 61–55 °C (decreasing 0.6 °C per cycle) and then 26 cycles of 94 °C for 20 s and 55 °C for 1 min. Additional cycling, which consisted of 94 °C for 20 s and 57 °C for 1 min (three cycles per step), was performed until tight genotyping clusters were obtained. The genotyping data were analyzed using StepOne v2.3.

### Agronomic trait evaluation

During consecutive growing seasons in 2017–2019, WTT11 and its recurrent parent Xiaoyan 81 were grown in triplicate in plots at the Xinxiang Experiment Station, Chinese Academy of Agricultural Sciences (113.5° E, 35.2° N). In each plot, 20 seeds were sown per 2.0-m row, with an inter-row spacing of 0.2 m. At physiological maturity, five whole plants from the center of the middle row were investigated for the following agronomic traits: plant height, effective tiller number, main panicle length, kernel number per main spike, thousand-kernel weight, and yield per plant. Excel and SPSS (v19.0) were used for statistical analyses.

## Results

### WTT11 carried a TTh·2DL translocation

**Fig. 2:**
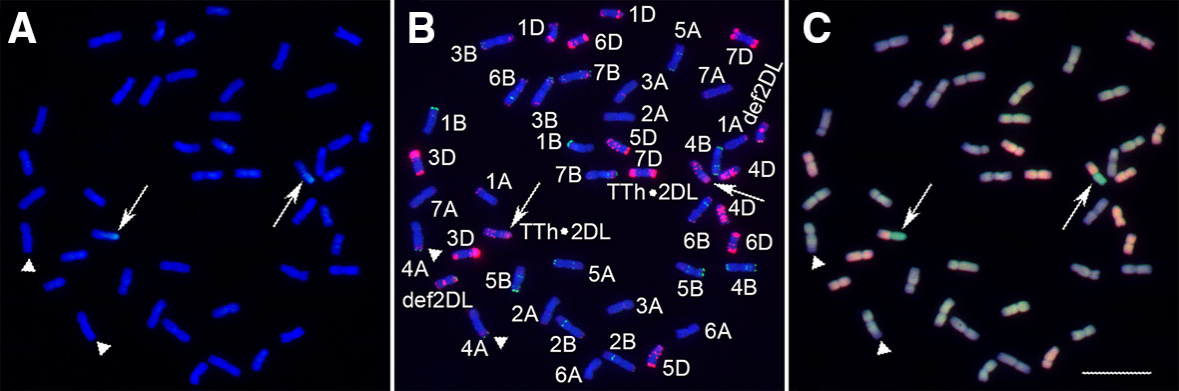
Genomic in situ hybridization (GISH), multi-color fluorescence in situ hybridization (mc-FISH) and multi-color GISH (mc-GISH) analyses of WTT11. GISH showed wheat chromosomes (blue) and *Th. intermedium* chromosome segments (green) (**A**). The mc-FISH showed the signal patterns of pAs1 (red) and pSc119.2 (green) in WTT11 (**B**). The mc-GISH showed the detection of chromosomes of the A-, B-, D- and *Th. intermedium* genomes based on yellow, blown, red and green fluorescence, respectively (**C**). The arrows note a pair of translocated chromosomes, and triangles note one pair of chromosomes 4A carrying B-genome chromosome segments. Bar = 20 μm

GISH analysis revealed that WTT11 carries 42 wheat chromosomes and two wheat–*Th. intermedium* whole-arm translocation chromosomes (Fig. 2A). In the mc-FISH analysis of translocation chromosomes, faint pAs1 signals were detected on the alien segments, and three pairs of strong punctate pAs1 signals were observed on the wheat fragments. One pair of strong punctate pAs1 signals appeared in the terminal regions of chromosome 2D. These results indicate that the long arm of chromosome 2D had broken and that the smaller segment had combined with the alien segment. The presence of the pAs1 and pSc119.2 signals demonstrate that WTT11 underwent a TTh·2DL translocation event (Fig. 2B). A mc-GISH analysis, which allowed A-, B-, D- and *Th. intermedium* genomes to be labeled with yellow, blown, red and green fluorescence, respectively, was performed to explore the genomic constitution of WTT11. This analysis revealed that WTT11 had 40 wheat chromosomes including 12, 14 and 14 from A, B and D genomes, respectively, plus one pair of interspecific translocation chromosomes and A–B translocation chromosomes. Similar to the Chinese Spring signal pattern, two small B-genome segments were found to be translocated in the terminals of 4AL (Fig. 2C).

### WTT11 was immune to stripe rust at seedling stage

**Fig. 3:**
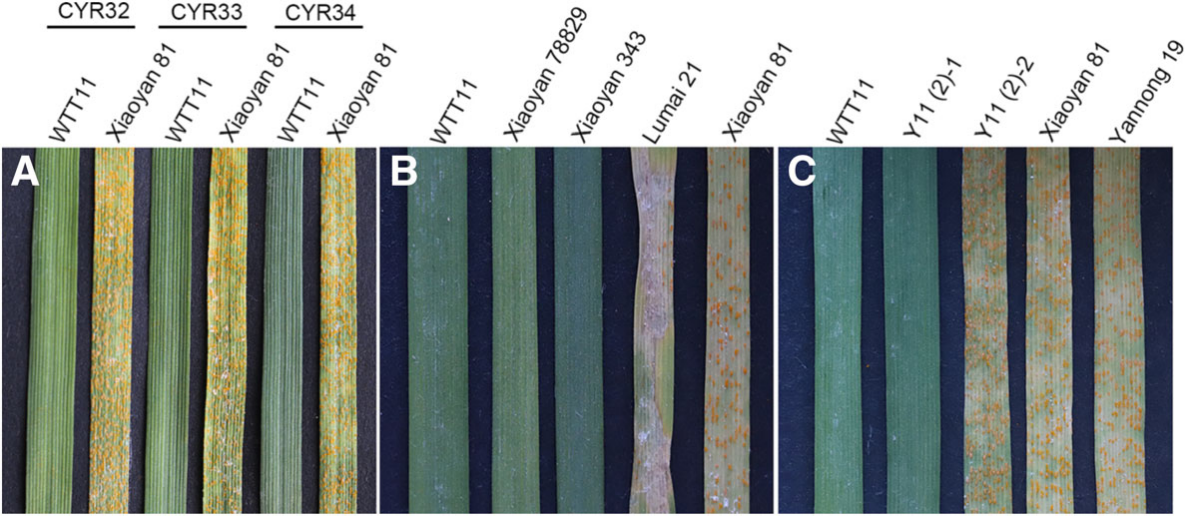
The seedling testing responses of WTT11 and its parents. Stripe rust responses of WTT11 and Xiaoyan 81 inoculated with *Pst* races CYR32, CYR33 and CYR34 (**A**). Stripe rust responses of WTT11 and its parents (**B**) and two individuals from Y11 (2) population (**C**) inoculated with *Pst* race CYR34. The seedling resistance evaluation results are shown: WTT11 (IT = 0;), Xiaoyan 78829 (IT = 0;), Xiaoyan 343 (IT = 0;), Lumai 21 (IT = 1), Xiaoyan 81 (IT = 3), Y11 (2)-1 (IT = 0), Y11 (2)-2 (IT = 3), Yannong 19 (IT = 3). Infection types 0–2 were resistance and 3–4 were susceptibility

Wheat cultivar Xiaoyan 81 was used as susceptible control and WTT11 was identified to immune to *Pst* races CYR32, CYR33 and CYR34 (Fig. 3A). Then, *Pst* race CYR34 was used to evaluate ITs of seedlings of the WTT11 translocation line and its parents. As shown in Fig. 3B, Xiaoyan 81 was susceptible to *Pst* race CYR34 (IT = 3). In contrast, WTT11 and its parents Xiaoyan 78829 and Xiaoyan 343 were nearly immune (IT = 0;), and Lumai 21 was highly resistant to CYR34 (IT = 1). To shed light on the origin of the stripe rust-resistance gene, we, therefore, performed backcrossing to transfer the alien segment from WTT11 into the CYR34-susceptible cultivar Yannong 19. A total of 121 individuals from the Y11 (2) population were screened by cytogenetic analyses and evaluation of strip rust resistance. A single alien segment signal was detected in 42 plants but was absent in the remaining 79. Analysis of resistance revealed that the 42 translocation individuals were immune (IT = 0), just like the resistant control WTT11, whereas the plants without the alien signal were as susceptible to infection (ITs = 3 or 4) (Fig. 3C; Table 1).

**Table 1:** Alien segment numbers and infect types of individuals in Y11 (2) population

Lines	Alien segment numbers	Infect types	Lines	Alien segment numbers	Infect types
Y11 (2)-1	1	0	Y11 (2)-62	1	0
Y11 (2)-2	0	3	Y11 (2)-63	0	4
Y11 (2)-3	1	0	Y11 (2)-64	0	4
Y11 (2)-4	0	3	Y11 (2)-65	0	3
Y11 (2)-5	1	0	Y11 (2)-66	0	4
Y11 (2)-6	0	3	Y11 (2)-67	0	3
Y11 (2)-7	0	3	Y11 (2)-68	1	0
Y11 (2)-8	0	3	Y11 (2)-69	0	4
Y11 (2)-9	0	3	Y11 (2)-70	0	4
Y11 (2)-10	0	3	Y11 (2)-71	0	4
Y11 (2)-11	1	0	Y11 (2)-72	1	0
Y11 (2)-12	0	3	Y11 (2)-73	1	0
Y11 (2)-13	0	3	Y11 (2)-74	0	3
Y11 (2)-14	1	0	Y11 (2)-75	0	3
Y11 (2)-15	1	0	Y11 (2)-76	0	3
Y11 (2)-16	0	3	Y11 (2)-77	0	3
Y11 (2)-17	0	3	Y11 (2)-78	0	4
Y11 (2)-18	0	3	Y11 (2)-79	0	4
Y11 (2)-19	1	0	Y11 (2)-80	0	4
Y11 (2)-20	0	3	Y11 (2)-81	0	4
Y11 (2)-21	0	3	Y11 (2)-82	0	3
Y11 (2)-22	1	0	Y11 (2)-83	0	3
Y11 (2)-23	1	0	Y11 (2)-84	1	0
Y11 (2)-24	0	3	Y11 (2)-85	0	3
Y11 (2)-25	1	0	Y11 (2)-86	0	3
Y11 (2)-26	1	0	Y11 (2)-87	0	4
Y11 (2)-27	0	3	Y11 (2)-88	1	0
Y11 (2)-28	0	3	Y11 (2)-89	1	0
Y11 (2)-29	1	0	Y11 (2)-90	1	0
Y11 (2)-30	0	3	Y11 (2)-91	1	0
Y11 (2)-31	1	0	Y11 (2)-92	1	0
Y11 (2)-32	1	0	Y11 (2)-93	1	0
Y11 (2)-33	1	0	Y11 (2)-94	0	3
Y11 (2)-34	1	0	Y11 (2)-95	0	4
Y11 (2)-35	0	3	Y11 (2)-96	0	4
Y11 (2)-36	1	0	Y11 (2)-97	0	4
Y11 (2)-37	0	3	Y11 (2)-98	1	0
Y11 (2)-38	1	0	Y11 (2)-99	0	3
Y11 (2)-39	1	0	Y11 (2)-100	0	3
Y11 (2)-40	1	0	Y11 (2)-101	0	3
Y11 (2)-41	0	3	Y11 (2)-102	0	3
Y11 (2)-42	1	0	Y11 (2)-103	0	4
Y11 (2)-43	0	3	Y11 (2)-104	0	4
Y11 (2)-44	0	3	Y11 (2)-105	0	4
Y11 (2)-45	0	3	Y11 (2)-106	0	4
Y11 (2)-46	0	3	Y11 (2)-107	1	0
Y11 (2)-47	0	3	Y11 (2)-108	0	4
Y11 (2)-48	0	3	Y11 (2)-109	0	4
Y11 (2)-49	0	3	Y11 (2)-110	0	4
Y11 (2)-50	0	3	Y11 (2)-111	0	3
Y11 (2)-51	0	3	Y11 (2)-112	0	3
Y11 (2)-52	0	3	Y11 (2)-113	1	0
Y11 (2)-53	0	3	Y11 (2)-114	1	0
Y11 (2)-54	1	0	Y11 (2)-115	0	3
Y11 (2)-55	1	0	Y11 (2)-116	0	3
Y11 (2)-56	0	3	Y11 (2)-117	0	3
Y11 (2)-57	1	0	Y11 (2)-118	0	4
Y11 (2)-58	0	4	Y11 (2)-119	1	0
Y11 (2)-59	1	0	Y11 (2)-120	0	4
Y11 (2)-60	0	4	Y11 (2)-121	0	4
Y11 (2)-61	1	0			

Infection types 0-2 indicate resistance and 3-4 susceptibility

### WTT11 might contain a novel stripe rust-resistance gene

Three reported diagnostic markers of *Th. intermedium* were used to amplify the genome of WTT11 and its parents. The amplification results are shown in Fig. S1. For *Xbarc1096*, the same DNA bands in WTT11 and its wheat parents were amplified, while no DNA bands in *Th. intermedium* were amplified, indicating WTT11 lacks *Yr50*. For *Xbarc187-1B-1*, linked with *YrL693*, the amplified products of WTT11 were different from those of *Th. intermedium*, implying WTT11 lacks *YrL693* as well. In addition, the SCAR marker *D05-A4-1* could amplify specific DNA band in *Th. intermedium*, but not in WTT11, indicating WTT11 does not contain *YrCH-1BS.*

### Deletion and homoeologous relationship were revealed

**Fig. 4:**
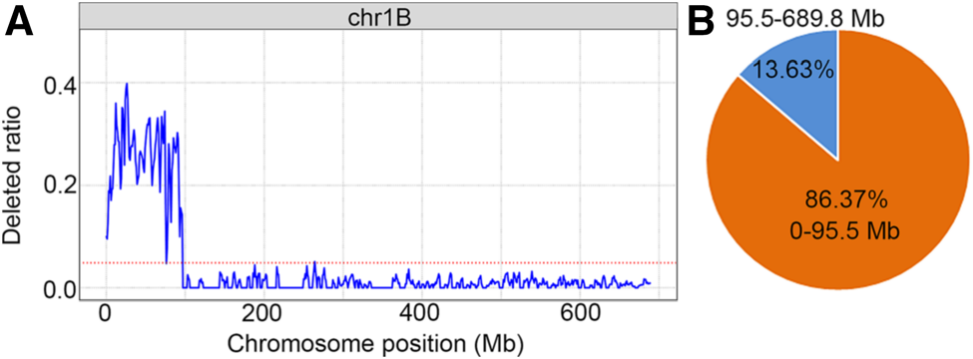
The deletion analysis in WTT11 genome. The deleted ratio for chromosome 1B (**A**). The red line indicated an average deleted value in chromosome 1B. The deleted ratio was calculated in 3-MB sliding windows, with 1-MB steps. The breakpoint of 1B was detected at 95.5 MB. The percentage of deleted SNPs distributed in two physical regions of chromosome 1B (**B**). Counting the percentage of the missing SNPs in each physical region of 1B, 86.37% of the missing SNPs on chromosome 1B were within the region 0–95.5 MB

Wheat660K SNP array analyses of WTT11, Xiaoyan 81 and *Th. intermedium* were carried out following a standard procedure. Out of 660,009 markers on the SNP array, 645,395 located at unique physical positions were selected to reveal deletion events in WTT11. Among them, 9,724 SNPs, excluding those undetected in three replicates of Xiaoyan 81, were absent in WTT11. As indicated by the distribution of SNP deletion ratios, the terminal region of chromosome arm 1BS was involved in a deletion event, and the breakpoint was detected at 95.5 MB (Fig. 4A). A count of missing SNPs on chromosome arm 1BS revealed that 1,489 SNPs were absent from chromosome arm 1BS, including 1,286 (86.37%) formerly present within the 0–95.5-MB region (Fig. 4B).

**Fig. 5:**
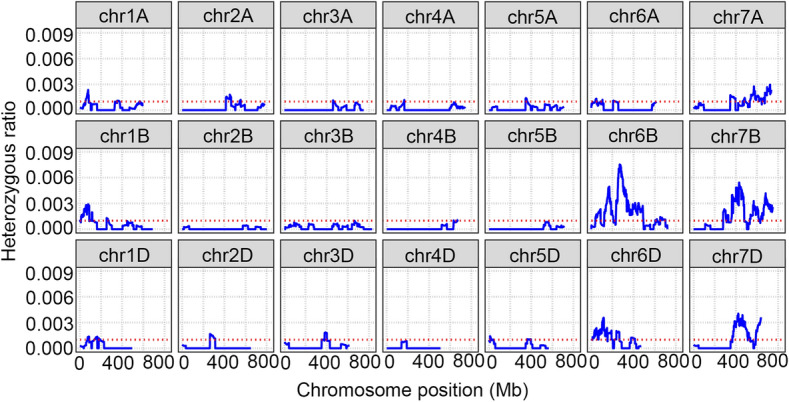
The homoeologous analysis of alien chromosomal segments from WTT11. The heterozygous ratio was heterozygous SNPs in WTT11, in accordance with both *Th. intermedium* and Xiaoyan 81’s genotypes, to all SNPs in each chromosome. It was counted in 50-MB sliding windows at 1-MB intervals, and plotted along the chromosome. The alien segments were homoeologous to wheat chromosome fragment with the highest ratio

To explore the homoeologous relationships of alien chromosomes in WTT11, we examined heterozygous genotypes while taking into account the results of the cytogenetic analyses. A total of 299 SNPs that were not only heterozygous but also consistent with the genotypes of both *Th. intermedium* and Xiaoyan 81 were selected and counted in 50-MB sliding windows at 1-MB intervals. As shown in Fig. 5, heterozygous SNPs were mainly distributed on chromosomes 6B, 6D, 7A, 7B and 7D, thus indicating that the alien segments were homoeologous to wheat-group chromosomes 6 and 7.

### Ten specific markers could trace alien chromosome segments of WTT11 effectively

**Fig. 6:**
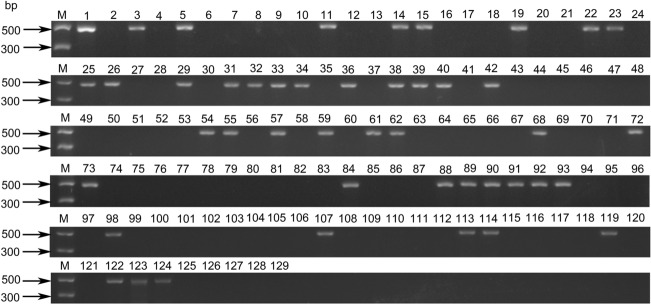
The amplification result of *M-XNXY68-151* in 121 individuals from Y11 (2) population and its parents. M: Marker II, 1–121: Y11 (2) 1–121, 122: WTT11, 123: *Th. intermedium*, 124: Xiaoyan 78829, 125: Xiaoyan 343, 126: Lumai 21, 127: Xiaoyan 81, 128: Yannong 19, 129: Chinese Spring

Ten specific markers for WTT11 were amplified in the 121 individuals from Y11 (2) population and its parents. The primer sequences and annealing temperatures are listed in Table S3. Agarose gel electrophoresis showed that markers *M-XNXY68-4*, *M-XNXY68-33*, *M-XNXY68-41*, *M-XNXY68-50*, *M-XNXY68-86*, *M-XNXY68-100*, *M-XNXY68-151*, *M-XNXY68-353*, *M-XNXY68-361* and *M-XNXY68-390* were able to amplify specific bands in WTT11, *Th. intermedium*, Xiaoyan 78829 and individuals harboring a single alien segment from Y11 (2) population, but did not amplify other individuals of the Y11 (2) population nor Xiaoyan 343, Lumai 21, Xiaoyan 81, Yannong 19 or Chinese Spring (Fig. 6). These amplification results were consistent with the results of the cytogenetic analyses and the resistance evaluation. These 10 markers were thus regarded as specific markers for the alien chromosomal segments of WTT11.

### KASP markers were developed to distinguish *Th. intermedium* and wheat genotypes

**Fig. 7:**
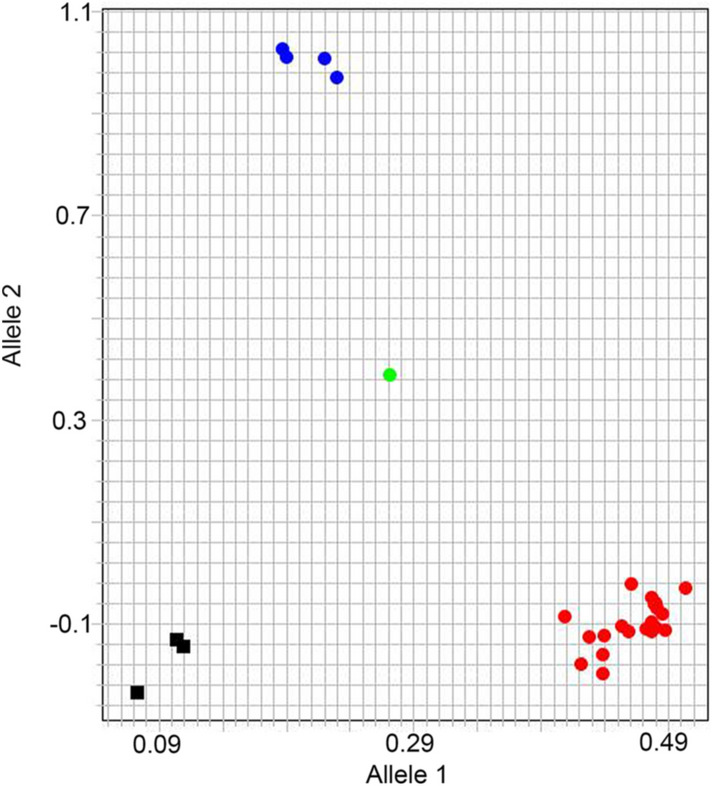
KASP marker amplification result of *Kasp-AX-109538133*. Using the KASP marker *Kasp-AX-109538133*, the genotyping result of 19 *Th. intermedium* accessions and 6 wheat cultivars was displayed. Red rotund shapes represent the homozygous 19 *Th. intermedium* accessions and Chinese Spring special SNPs, blue rotund shapes represent the homozygous common wheat special SNPs and green rotund shape represents the heterozygous Jimai 22 special SNP

Drawing on the results of the SNP microarray analysis, flanking sequences of 299 SNP markers were compared against the wheat reference sequence (IWGSC RefSeq v1.0) in a BLASTN search. A total of 60 (20.07%) SNP flanking sequences were present in only one copy in the wheat genome, and corresponding primer pairs were designed using the KASP primer design platform. The results of amplifications using these primers suggested that 20 KASP markers could differentiate *Th. intermedium* and common wheat genotypes (Table S4). For example, amplification with *Kasp-AX-109538133* revealed that 19 *Th. intermedium* accessions and Chinese Spring had C/C alleles, while Xiaoyan 343, Lumai 21, Xiaoyan 81 and Yannong 19 possessed T/T alleles, and Jimai 22 carried C/T alleles (Fig. 7).

### WTT11 possessed excellent agronomic performance

The phenotypic characteristics of WTT11 and its recurrent parent Xiaoyan 81 were investigated during two consecutive seasons (Table 2). Compared with Xiaoyan 81, WTT11 plants had significantly lighter thousand-kernel weights (*P* < 0.01), but longer main panicles and more kernel number per main spike. It was remarkable that yield per plant of WTT11 and Xiaoyan 81 was similar and did not show any significant difference in both growing seasons. Besides, WTT11 plants had significantly lower (*P* < 0.01) plant height than Xiaoyan 81 in 2018–2019 years.

**Table 2:** The agronomic performance of WTT11 and Xiaoyan 81 in two consecutive seasons 2017–2019

Years	Lines	Plant height (cm)	Effective tiller number	Main panicle length (cm)	Kernel number per main spike	Thousand-kernel weight (g)	Yield per plant (g)
2018	WTT11	59.30 ± 3.60	10.80 ± 2.59	8.34 ± 0.44	54.00 ± 4.58	29.51 ± 1.88	11.07 ± 2.66
	Xiaoyan 81	62.70 ± 1.89	9.40 ± 1.52	7.74 ± 0.53	51.40 ± 9.53	37.00 ± 1.51**	13.07 ± 1.90
2019	WTT11	63.70 ± 1.04	15.40 ± 1.67	9.18 ± 0.49	57.40 ± 5.59	41.73 ± 1.49	20.08 ± 2.71
	Xiaoyan 81	82.00 ± 2.74**	16.60 ± 3.05	8.80 ± 0.45	47.40 ± 5.27	48.89 ± 0.63**	21.78 ± 2.33

*Significant difference determined at *P* < 0.05, **significant difference determined at *P* < 0.01

## Discussion

### A linkage analysis was conducted to explore the origin of disease resistance in WTT11

Pedigree derivation has frequently been used to confirm the origin of resistance genes in wheat–alien introgressions. For instance, the wheat–rye 4R chromosome disomic addition line WR35 was developed by crossing Xiaoyan 6 with the rye cultivar German White. At the seedling stage, evaluation of stripe rust reactions revealed that German White is immune or nearly immune to *Pst* races CYR31, CYR32, CYR33 and CYR34 (IT = 0 or 0;), whereas WR35 is highly resistant (IT = 1) and Xiaoyan 6 is susceptible (IT = 4) to all four races. These results suggest that WR35 possesses a new stripe rust-resistance gene that differs from resistance genes known from rye (An et al. 2019). As another example, the partial amphiploid TAI7047 was derived from hybrids of *Th. intermedium* and common wheat Jinchun 5 and Taiyuan 768. The wheat introgression line CH223 was then developed by crossing TAI7047 with common wheat Jing 411 and Jinmai 33. Evaluation of stripe rust resistance revealed that CH223 and TAI7047 are both immune and nearly immune to races CYR32, CYR33 and CYR34, while Jinchun 5, Taiyuan 768, Jing 411 and Jinmai 33 are all susceptible to CYR30, CYR31, CYR32 and CYR33. The stripe rust resistance of CH223 is thus presumably derived from *Th. intermedium* (Chang et al., 2010). For germplasm resources with known family trees, pedigree analysis is a convenient, feasible method for tracing disease-resistance inheritance.

Different from the above studies, some wheat parents of wheat–alien progeny are immune or highly resistant, or some are difficult to obtain for resistance evaluation. In this study, Xiaoyan 343, one of the parents of WTT11, was immune to stripe rust race CYR34. Lumai 21 was highly resistant to CYR34. Besides, NPFP, the wheat parent of partial amphiploid Xiaoyan 78829, was so antique that we could not retain it for resistance screening. Therefore, we used variety Yannong 19 that was susceptible to CYR34 as recurrent parent and constructed BC_2_F_1_ population to transfer the alien segments in its background. GISH and stripe rust evaluation results verified each other and determined obviously that the resistance gene was derived from *Th. intermedium*. Furthermore, three markers linked to *Yr50*, *YrL693* and *YrCH-1BS* did not amplify specific bands in WTT11. The wheat660K SNP array data indicated that alien chromosomal segments of WTT11 belong to the homoeologous group 6 and 7. Our studies suggested that the resistance of WTT11 originates from a novel gene derived from *Th. intermedium*.

### The wheat660K SNP array may be a more precise tool for physical mapping

Since homoeologous pairing and recombination have not taken place between alien and common wheat chromosomes, physical mapping is potentially an alternative way to map important genes on alien chromosomes. For example, Li et al. (2016) identified rye 6R and 6RL deletion lines and then localized a powdery mildew resistance gene by combining mc-FISH analysis with resistance assessment. Unfortunately, only a few FISH probes have been developed from alien genomes. In addition, exactly defining alien segment sizes, particularly those related to intercalary translocations and introgressions, is difficult to accomplish solely by cytogenetic methods. Many recent studies have, therefore, resorted to known genome sequences, such as those of *T. urartu*, *Ae. tauschii* and *Triticum aestivum*, to map alien genes more precisely. Dai et al. (2020) constructed a fine cytological map of 4VS by cytogenetic and 4VS-specific marker analysis. They carried out a BLASTN search of 199 marker sequences against the *Ae. tauschii* 4DS reference genome sequence and further assigned 39 bins to corresponding genome regions of chromosome arm 4DS; they then narrowed down the WYMV resistance gene *Wss1* locus to the corresponding physical region, i.e., a 0–14.3-MB physical distance on 4DS of *Ae. tauschii*. Many studies have revealed the existence of homoeology between the *Thinopyrum* genome and the wheat genome (Grewal et al. 2018; Cseh et al. 2019; Baker et al. 2020; Wang et al. 2020b). At the same time, several researchers have started to develop useful markers by comparative genomic analysis. For example, Guo et al. (2015) used sequences in the distal region of *Ae. tauschii* 7DL and a bread wheat array to develop SSR and DArT markers for 7el, an accomplishment that greatly contributed to the successful cloning of *Fhb7*. In addition, Zhang et al. (2019b) developed a series of closely linked PCR-based markers for *Sr26* based on comparative genomic analysis of NLR genes. In view of the high degree of homoeology between wheat and *Thinopyrum* genomes, future studies should rely more heavily on the wheat reference genome.

Using the wheat660K SNP array, 299 specific SNPs were located on alien segments in this study. Our analysis indicated that the alien segments are homoeologous with wheat-group chromosomes 6 and 7. Since cloned stripe rust-resistance genes are mostly NLR genes, resistance gene analogs (RGA) family annotation indicated that 286 and 354 RGA candidates were predicted in *Th. elongatum* chromosome 6E and 7E by the RGAugury pipeline (Wang et al. 2020b). To map the resistance gene in WTT11 and reduce deleterious linkage drag, many translocation lines with smaller alien segments need to be produced by ^60^Co γ-ray irradiation of WTT11. Depending on the physical positions of the specific SNPs, a fine physical map for alien segments of WTT11 can then be constructed.

### All detection methods are equally important in wheat chromosome engineering

Cytogenetic analyses, such as GISH, mc-FISH and mc-GISH, mainly explore aspects such as types of chromosome structural variation, numbers and sizes of alien chromosomes or chromosome fragments, and genomic constitution. Since their results are clearly visible, GISH and FISH are the first methods of choice when analyzing germplasm developed by chromosome engineering. Many partial amphiploids and wheat–alien addition, substitution and translocation lines have been successfully analyzed by these molecular cytogenetic techniques (Zhang et al. 1996; Benavente et al. 1996; Friebe et al. 2000; Nagy et al. 2002; Malysheva et al. 2003; Han et al. 2004).

Since GISH and FISH are costly, labor intensive and time consuming, however, many markers specific for alien chromosomes, such as random amplified polymorphic DNAs markers (King et al. 1993), sequence-tagged markers (Luan et al. 2010), PCR-based landmark unique gene markers (Ardalani et al. 2016), IT markers (Wang et al. 2017), and SNP markers (Tiwari et al. 2014), have been developed to easily identify alien chromosomes in a wheat background. The SLAF-seq technique enables the efficient and convenient development of specific PCR-based markers for plant species with uncharacterized genomes. An increasing number of SLAF markers are, therefore, being applied in wheat chromosome engineering to rapidly trace alien segments. For example, 67 *Th. ponticum*-specific SLAF markers were developed from the tiny wheat–*Th. ponticum* translocation line EA and subsequently verified in the F_2_ population of a cross between wheat cultivar Xiaoyan 60 and EA (Liu et al. 2018).

Stable homozygous introgressions are easily analyzed and can, therefore, obviously be applied to some characters in wheat breeding. Most PCR-based molecular markers are dominant, however, which limits their ability to distinguish between heterozygous and stable homozygous introgressions. KASP markers, which constitute a novel type of co-dominant molecular markers, have fortunately been developed and can rapidly detect alien segments and provide information on their homozygosity (Grewal et al. 2020a). Many putative SNPs based on SNP array, RNA-seq and SLAF-seq analyses have been converted into KASP markers to allow discrimination of alien chromosomes from the wheat genome (Zhou et al. 2017; Grewal et al. 2020b; Han et al. 2020). In our studies, we have, therefore, developed specific KASP markers to distinguish *Th. intermedium* and wheat for subsequent application in homozygous germplasm screening.

In summary, a new wheat–*Th. intermedium* translocation line, WTT11, was produced by distant hybridization and assessed for immunity to *Pst* races CYR32, CYR33 and CYR34. Cytogenetic analyses revealed that the alien segments were translocated into the long arm of chromosome 2D. A wheat660K SNP array analysis also suggested that a deletion occurred in the 0–95.5-MB region of chromosome 1B. At the same time, the alien segments were homoeologous to wheat-group chromosomes 6 and 7. Furthermore, 10 specific PCR-based markers for alien segments and 20 KASP markers distinguishing *Th. intermedium* and wheat were developed. These new markers can be used in wheat breeding programs to effectively trace alien segments conferring stripe rust resistance.

## Supplementary Information

Below is the link to the electronic supplementary material.

Supplementary file1 (DOCX 1057 kb)

Supplementary file2 (XLSX 9 kb)

Supplementary file3 (XLSX 9 kb)

Supplementary file4 (XLSX 11 kb)

Supplementary file5 (TIF 30677 kb)
