# Supplementary figures and images for: Cytogenetic identification and molecular marker development for the novel stripe rust-resistant wheat–Thinopyrum intermedium translocation line WTT11
Source: aBIOTECH. 2021 Oct 11;2(4):343–56. doi: 10.1007/s42994-021-00060-3 (PMC9590478; doi:10.1007/s42994-021-00060-3)

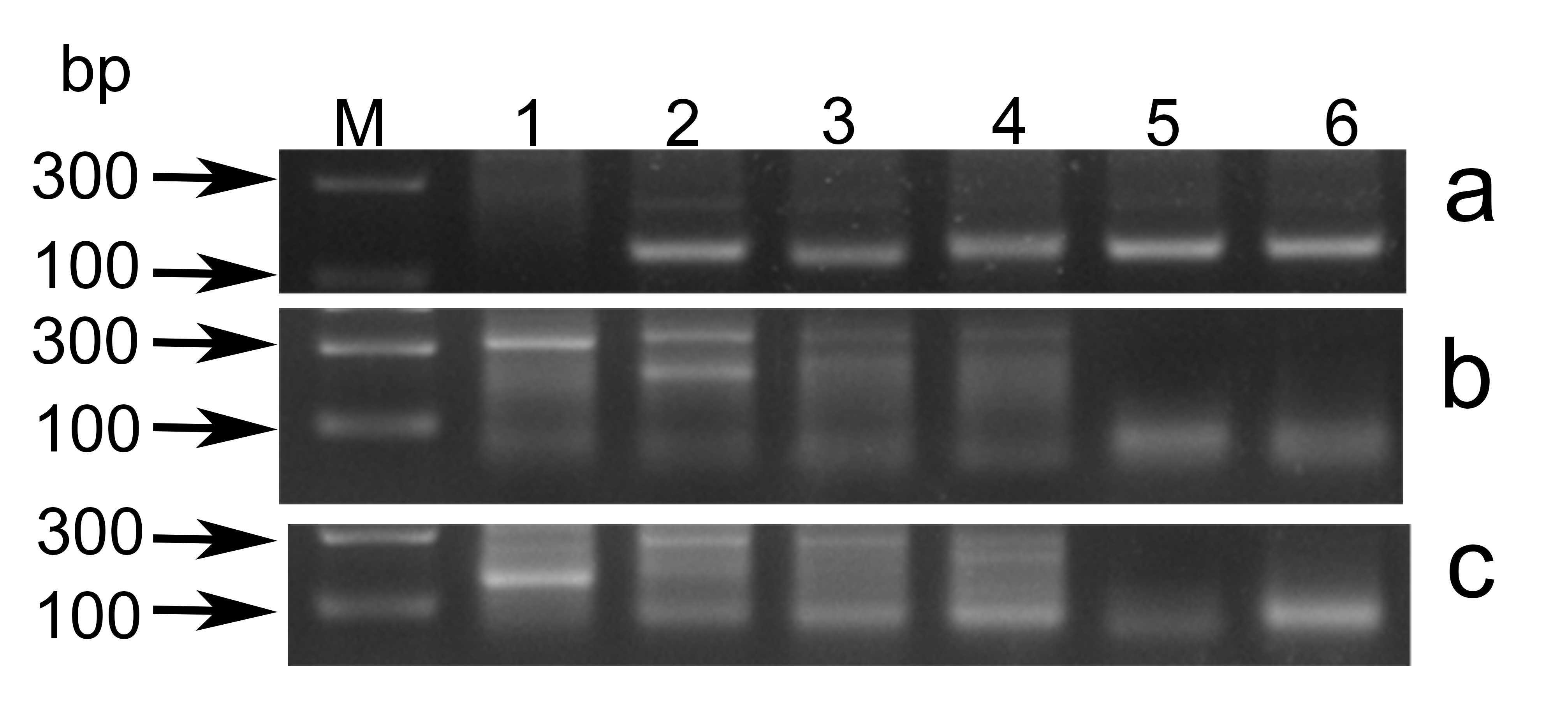

Supplement: Supplementary file 5 — Supplementary file5 (TIF 30677 kb) [file 42994_2021_60_MOESM5_ESM.tif]
